# Construction of a Stable and Temperature-Responsive Yeast Cell Factory for Crocetin Biosynthesis Using CRISPR-Cas9

**DOI:** 10.3389/fbioe.2020.00653

**Published:** 2020-06-30

**Authors:** Tengfei Liu, Chang Dong, Mingming Qi, Bei Zhang, Lei Huang, Zhinan Xu, Jiazhang Lian

**Affiliations:** ^1^Key Laboratory of Biomass Chemical Engineering of Ministry of Education, College of Chemical and Biological Engineering, Zhejiang University, Hangzhou, China; ^2^Center for Synthetic Biology, College of Chemical and Biological Engineering, Zhejiang University, Hangzhou, China; ^3^School of Bioengineering, Dalian University of Technology, Dalian, China

**Keywords:** crocetin, temperature switch, copy number, genome integration, CRISPR-Cas9

## Abstract

Crocetin is a plant natural product with broad medicinal applications, such as improvement of sleep quality and attenuation of physical fatigue. However, crocetin production using microbial cell factories is still far from satisfaction, probably due to the conflict between cell growth and product accumulation. In the present work, a temperature-responsive crocetin-producing *Saccharomyces cerevisiae* strain was established to coordinate cell growth, precursor (zeaxanthin) generation, and product (crocetin) biosynthesis. The production of crocetin was further enhanced via increasing the copy numbers of *CCD2* and *ALDH* genes using the CRISPR-Cas9 based multiplex genome integration technology. The final engineered strain TL009 produced crocetin up to 139.67 ± 2.24 μg/g DCW. The advantage of the temperature switch based crocetin production was particularly demonstrated by much higher zeaxanthin conversion yield. This study highlights the potential of the temperature-responsive yeast platform strains to increase the production of other valuable carotenoid derivatives.

## Introduction

Crocetin (C_20_H_24_O_4_) has been found in the stigmas of *Crocus sativus* L. and the fruit of *Gardenia jasminoides* (Sheu and Hsin, 1998; Frusciante et al., 2014) and contributes to the most important therapeutic effects of saffron (Hashemi and Hosseinzadeh, 2019). Crocetin has different pharmacological effects on a large number of cancer cells: liver, ovarian, breast, prostate, leukemia, colorectal, bladder, lung, tongue carcinoma, and esophageal (Colapietro et al., 2019). The retail price of the red stigmas of *C. sativus* ranges from 2,000 to 7,000 £/kg, because 1 kg of dry saffron requires the manual harvest of around 110,000–170,000 flowers (Frusciante et al., 2014). As an apocarotenoid, crocetin is isolated from the saffron stigmas and large-scale plantation of saffron crocus is required for commercial applications. Alternatively, microbial production of carotenoids and their derivatives have been demonstrated as a promising solution (Niu et al., 2017; Wang C. et al., 2019). Therefore, *de novo* biosynthesis of crocetin from carbohydrates using microbial cell factories would be a more sustainable and economic way.

**Figure 1:**
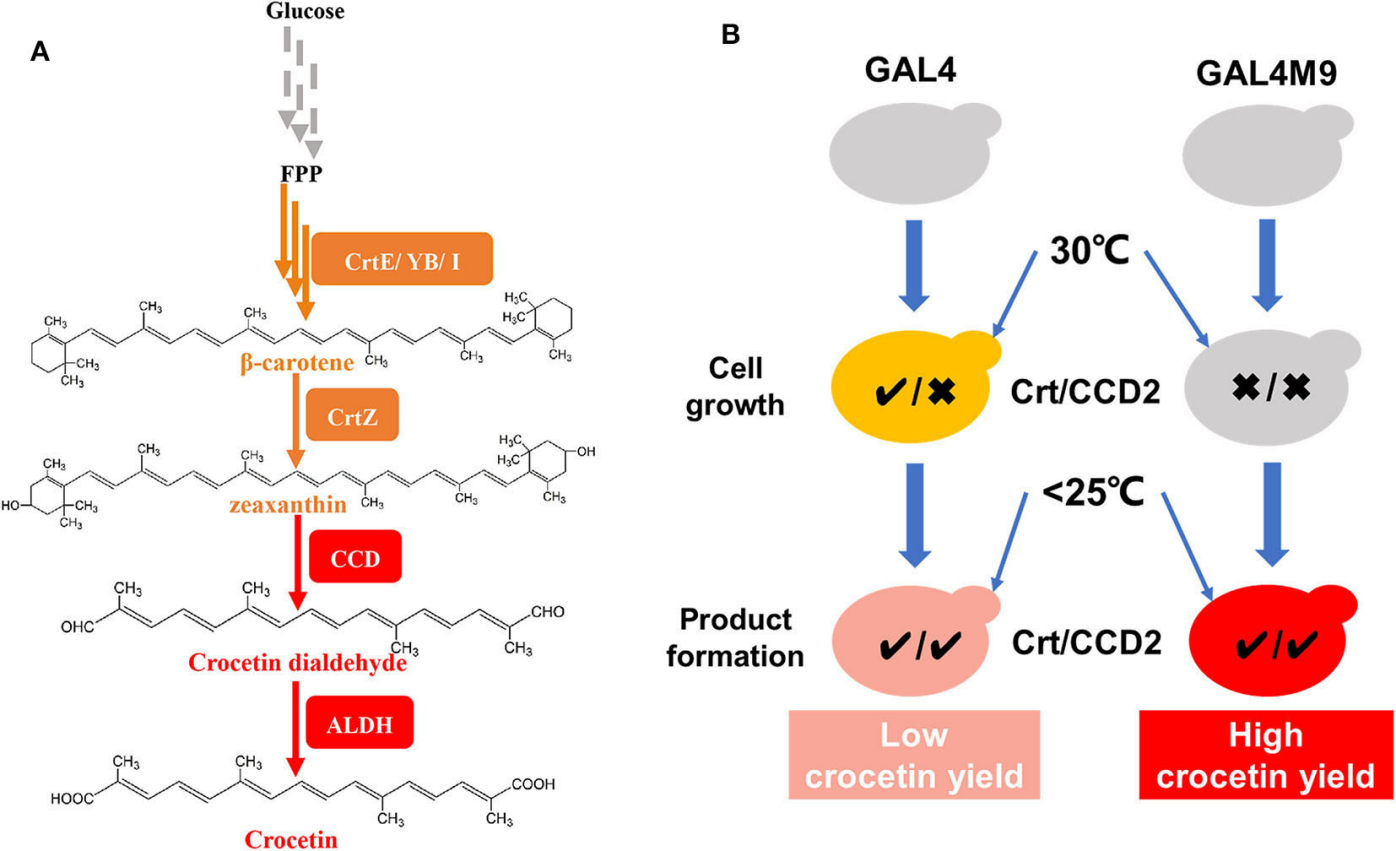
Overview on the construction of a temperature-responsive yeast cell factory for crocetin biosynthesis. **(A)** *De novo* crocetin biosynthetic pathways from glucose. The carotenogenic genes were shown in orange and the crocetin biosynthetic pathway genes were shown in red. **(B)** The strategy of GAL4M9 based temperature switch to synchronize zeaxanthin biosynthesis (denoted by the activity of Crt) and cleavage (denoted by the activity of CCD2, equivalent to crocetin biosynthesis). The traditional two-stage fermentation strategy (left panel) resulted in physical separation of the substrate zeaxanthin (efficient synthesis at 30°C and storage at the cellular membranes) the CCD2 enzyme (functional at lower temperatures), accordingly low zeaxanthin conversion yield. In the temperature-responsive strain, zeaxanthin biosynthesis and CCD2 functional expression were synchronized at the lower temperature stage and higher zeaxanthin conversion yield could be achieved. CrtE, lycopene ε-cyclase; CrtYB, bifunctional phytoene synthase and lycopene cyclase; CrtI, phytoene desaturase; CrtZ, β-carotene hydroxylase; CCD, carotenoid cleavage dioxygenase; ALDH, aldehyde dehydrogenase; GAL4, transcription factor for activating GAL regulon genes; GAL4M9, GAL4 mutant showing low temperature induction.

The biosynthesis of crocetin in *C. sativus* stigmas starting from β-carotene contains three major steps, catalyzed by a β-carotene hydroxylase (CrtZ), a carotenoid-cleaving dioxygenase (CCD2), and an aldehyde dehydrogenase (ALDH), respectively (Figure 1A; Frusciante et al., 2014). The introduction of these three genes together with the carotenogenic genes enabled the production of crocetin in engineered *Saccharomyces cerevisiae* (Chai et al., 2017; Tan et al., 2019) and *Escherichia coli* (Wang W. et al., 2019) strains. Chai et al. found that the production of crocetin was much higher at lower temperature than at 30°C (the optimal temperature for cell growth), probably due to the higher enzymatic activity of CCD2 from *C. sativus* L (Ahrazem et al., 2016). To address the dilemma between cell growth and product formation, a general strategy is to perform two-stage fermentation, shifting culture temperature from 30°C to a lower level (i.e., 25 or 20°C) when the cell density reaches to a relatively high level. Although high-level production of crocetin was achieved using such a strategy, the conversion yield of zeaxanthin to crocetin remained at a low level. As a lipophilic and water-insoluble compound (Murill et al., 2019), zeaxanthin was synthesized and stored in the cellular membranes of microorganisms (Shen et al., 2016; Sun et al., 2018). In this case, due to low CCD2 enzymatic activity at 30°C, zeaxanthin was synthesized at high efficiency and mainly accumulated in the cellular membranes in *S. cerevisiae* (Chai et al., 2017). Although the activity of CCD2 was significantly enhanced by shifting to low temperature, the physical separation of the enzyme (present in the cytoplasm) and the substrate (storage in the cellular membranes) in two compartments led to low zeaxanthin cleavage and conversion efficiency (Figure 1B). Therefore, how to balance zeaxanthin accumulation and temperature-regulated CCD2 activity became a key question to enhance zeaxanthin conversion and crocetin production.

The use of fermentation temperature as a general input signal for gene expression regulation has a number of advantages including ready controllability, fast temporal response, high reversibility, and wide applicability (Chakshusmathi et al., 2004). Therefore, the construction of a temperature-responsive cell factory is a promising approach for crocetin production. In previous studies, a few temperature-induced protein expression systems have been established in yeast, such as those based on the mutation of the acid phosphatase regulatory genes *PHO80* and *PHO4*^ts^ (Kramer et al., 1984), mating type control involving *SIR3* mutation and *MAT*α2-hybrid promoters (Sledziewski et al., 1990), as well as the modified GAL regulation system (Xie et al., 2014; Zhou et al., 2018). Among these nicely designed systems, the GAL regulon based system demonstrated the advantages of easy manipulation (single point mutation of the GAL4 activator) and high expression level of heterologous genes (>1,000-fold induction). The GAL based temperature switch was established by knocking out *GAL80* encoding the *GAL4* inhibitor and replacing the wild-type *GAL4* with the temperature-sensitive mutant *GAL4M9*. The evolved GAL4M9 (a single point mutation of GAL4) enabled the expression of the GAL regulon to be only turned on at lower temperatures, with 24°C determined to be the optimal induction temperature (Zhou et al., 2018). The application of the temperature switch was demonstrated by the production of lycopene and astaxanthin with temperature as an input signal for metabolic pathway regulation in yeast cell factories (Zhou et al., 2018, 2019).

In the present study, the temperature-responsive yeast cell factory was evaluated for the application in the biosynthesis of crocetin, by coordinately regulated the biosynthesis and cleavage of zeaxanthin in a temperature-dependent manner. Firstly, the temperature-responsive yeast strain was reconstructed by knocking out *GAL4* and *GAL80*, followed by the introduction of *GAL4M9* expression cassette (Figure S1). Then the crocetin biosynthetic pathway genes under the control of GAL promoters were integrated into the chromosome, using the CRISPR-Cas9 technology (Lian et al., 2018a,b), to create a stable and temperature-responsive yeast strain for crocetin production. Thanks to the high efficiency of genome integration, crocetin biosynthesis was further optimized by integrating different copy numbers of *CCD2* and *ALDH* genes. Finally, a temperature-responsive and stable yeast strain was established to produce crocetin with a titer of 139.67 ± 2.24 μg/g DCW and a zeaxanthin conversion yield of up to 77%.

## Materials and Methods

### Strains, Media, and Chemicals

*E. coli* Trans T1 (Transgen Biotech Co., Ltd., Beijing, China) was use for gene cloning and plasmid amplification. Recombinant *E. coli* strains were cultured in LB medium (OXIOD Bio-tech Co., Ltd., London, England) supplemented with 100 mg/L ampicillin. *S. cerevisiae* BY4741 strain was used as the host for genome engineering and crocetin production. Yeast strains were routinely cultivated in YPD medium (OXIOD). Synthetic complete medium (SCD) containing 5 g/L ammonium sulfate, 1.7 g/L yeast nitrogen base without ammonium and amino acids (BD Diagnostics), 0.6 g/L CMS missing the appropriate nutrients, and 20 g/L glucose. When necessary, 200 mg/L G418 sulfate (Sangon Bio-tech Co., Ltd., Shanghai, China) was supplemented. All chemicals were bought from Sigma-Aldrich (St. Louis Missouri, USA), unless specifically mentioned. Crocetin (Figure S2A) and zeaxanthin (Figure S2B) standards were purchased from Yuanye Bio-tech Co., Ltd. (Shanghai, China) and Chemface Bio-tech Co., Ltd. (Wuhan, China), respectively.

### Plasmid and Strain Construction

KOD-Plus-Neo DNA Polymerase (TOYOBO Biotech Co., Ltd., Tokyo, Japan) was used for gene amplification and PCR products were purified by the Gene JET PCR Purification Kit (Thermofisher Scientific, Shanghai, China). Restriction enzymes and T4 DNA ligase were purchased from NEB (Beijing, China). Plasmids were extracted from *E. coli* using the AxyPrep Plasmid Miniprep Kit (Axygen) according to manufacturer's instructions. DNA sequencing was performed by Tsingke Biotech Co., Ltd. (Hangzhou, China).

*CrtI* (accession number: Y15007.1), *CrtYB* (accession number: KJ783314.1), and *CrtE* (accession number: DQ016502.1) were amplified from the genomic DNA of the yeast strain CEN-Crt and *CrtZ* (accession number: D90087.2) was amplified from pRS426-Zea (Lian et al., 2016, 2017). CCD2 from *C. sativus* L. (*CsCCD2*; accession number: KJ541749.1) and *ALDH*s (Trautmann et al., 2013; Costantina et al., 2018) from *Synechocystis* sp. PCC6803 (*syaldh*; accession number: WP_010873792) and *C. sativus* L. (*CsALDH*; accession number: MF596165.1) were codon optimized and synthesized by Tsingke Biotech (Table S1). All these genes were cloned into the multiple cloning sites (MSCs) of the pESC vectors (Figure S3), pESC-URA, pESC-LEU, and pESC-LEU2d by restriction digestion/ligation (MCS1: *Bam*HI/*Xho*I; MCS2: *Not*I) or Gibson Assembly.

Gene deletion and integration in *S. cerevisiae* were performed using the CRISPR-Cas9 method (Lian et al., 2017) and the schematic overview was briefly demonstrated in Figure S4. The guide RNA (gRNA) sequences were designed using the Benchling CRISPR-Cas9 tool (https://www.benchling.com/crispr) and cloned into p423-SpSgH and p426-SpSgH, constructed in our previous studies (Lian et al., 2017, 2019). For the construction of strains TL001-TL014, the expression cassettes were amplified by PCR containing 40 bp homology arms to the target chromosomal locus and co-transformed with the corresponding gRNA plasmid to the yeast strains using the LiAc/SS carrier DNA/PEG method (Gietz and Schiestl, 2007). The strains and plasmids used in this study were listed in Table S2, the corresponding primers were listed in Table S3, and the gRNA sequences as well as the chromosome loci for the integration of the heterologous gene expression cassettes were listed in Table S4.

### Fermentation Conditions

For crocetin quantification, a single colony was picked from YPD or SCD agar plates and subcultured in tubes at 30°C and 250 rpm until saturation. Then, 300 μL seed culture was inoculated into a 250 mL flask containing 30 mL YPD (for the chromosome integrated strains) or SCD (for the plasmid bearing yeast strains). After culturing at 30°C for 24 h, temperature was shifted to 24 or 20°C and fermentation was continued for additional 7–8 days. All the experiments were performed in biological triplicates.

### Carotenoid Extraction

Carotenoids were extracted from yeast cells according to the previous protocol (Chai et al., 2017). Three milliliter cells were harvested by centrifugation at 12,000 rpm for 3 min, washed with 3 mL distilled water, and suspended in 0.5 mL of 3M HCl. The suspension was boiled for 2 min and chilled on ice immediately for 3 min. The cell pellet was resuspended in 100 μL of 50:50 MeOH: acetone containing 1% (w/v) butylated hydroxytoluene and each sample was extracted twice with the same amount of solvent.

### HPLC-MS Quantification

The supernatant was passed through a 0.22 μm membrane filter and directly analyzed using a SHIMADZU Liquid chromatography-tandem mass spectrometry (LC-MS/MS 8045) equipped with a UV detector at 420 nm and 30°C. Separation of compounds were performed on a HyPURITY^TM^ C18 HPLC column (150 mm × 4.6 mm, 3 μm, Thermo Scientific) with a flow rate of 0.5 mL/min. The mobile phase consisted of 10 mM ammonium formate solution (solvent A) and methanol (solvent B). The following gradient elution program was used: 60–2% solvent A over 20 min and returned to 60% solvent A over 20 min. The mass spectrometer was an APCI ion source equipped with a triple quadrupole mass analyzer and the negative ionization mode was used for carotenoid and crocetin analysis. The mass spectrometer was scanned from m/z 50 to 800. The desolvation line (DL) temperature was held at 200°C, with a spray voltage of 1.8 kV and an atomizing gas flow rate of 6 L/min.

## Results

### Construction of a Temperature-Regulated Crocetin Biosynthetic Pathway

**Figure 2:**
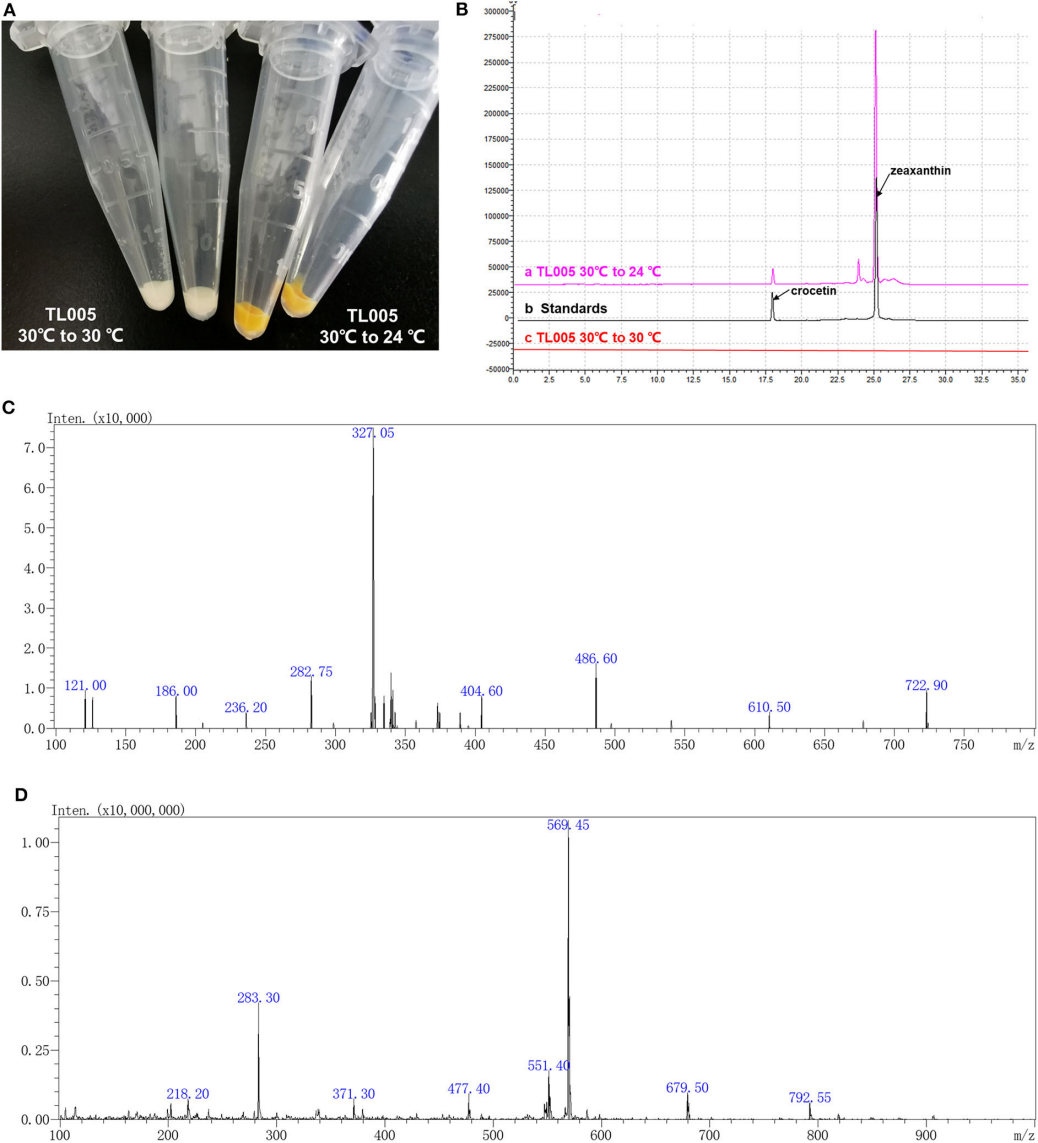
Temperature dependence of zeaxanthin and crocetin biosynthesis in the engineered yeast cell factory. **(A)** Color of the cell pellets (strain TL005) with (right two tubes, yellow-to-orange color) or without (left two tubes, no visible color) shifting temperature to 24°C. **(B)** HPLC chromatograms of the fermentation profiles of TL005, together with the crocetin and zeaxanthin standards. **(C)** MS spectra of crocetin produced by TL005. **(D)** MS spectra of zeaxanthin produced by TL005.

According to the previous study (Zhou et al., 2018), a temperature-responsive yeast strain with an engineered GAL regulon should be reconstructed by knocking out *GAL80* and *GAL4* and introducing the *GAL4M9* expression cassette. In order to construct a temperature-responsive crocetin producing strain (TL005), four carotenogenic genes (*CrtE, CrtYB, CrtI*, and *CrtZ*), crocetin biosynthesis genes (Cs*CCD2* and Cs*ALDH*), and *GAL4M9* was integrated to the yeast genome, together with the inactivation of *GAL4* and *GAL80* (Figure S1). More specifically, *GAL80* locus was replaced by the Cs*CCD2* and *CrtE* expression cassettes, *GAL4* locus was replaced by the *CrtZ* and Cs*ALDH* expression cassettes, followed by the integration of *CrtYB* and *CrtI* expression cassettes as well as the *GAL4M9* expression cassette. To verify the temperature-dependence of zeaxanthin and crocetin biosynthesis, strain TL005 was cultured with or without a temperature shift to 24°C. As shown in Figure 2A, no visible color was observed when the strain was constantly maintained at 30°C (Table S2, strain TL005), while yellow-to-orange pigment was formed after temperature switch, indicating a temperature-dependent biosynthesis of carotenoids. The biosynthesis of zeaxanthin and crocetin was further confirmed using HPLC-MS analysis (Figure 2B). Consistent with the pigment formation results, crocetin, and zeaxanthin production was only detected in strain TL005 after the temperature shift. A peak with a retention time (t_R_) of 17.85 min was identified as crocetin (m/z = 327.05) by MS (Figure 2C, Figure S2A for crocetin standard). Similarly, the biosynthesis of zeaxanthin (t_R_ = 25.02 min, m/z = 569.25) was also found to be temperature-dependent (Figure 2D, Figure S2B for zeaxanthin standard). Therefore, the temperature-responsive crocetin-producing yeast cell factory was successfully constructed.

### Optimization of Crocetin Biosynthesis by Adjusting Copy Numbers of *CCD2*-*ALDH* Genes

Although the production of crocetin was achieved, zeaxanthin was accumulated to high levels, indicating CCD2 as a rate-limiting enzyme for crocetin biosynthesis. Integrating multiple copies of the biosynthetic genes or pathways into the yeast genome has been reported to benefit the production of the target compounds (Li et al., 2015). In addition, in several cases, the chromosome integrated strains were found to demonstrate higher stability and accordingly higher production than multi-copy plasmid bearing yeast strains (Lee and Silva, 1997; Shi et al., 2014, 2016). SyAldh was found to enable efficient production of crocetin in a previous report (Chai et al., 2017) and was included for evaluation in the present study as well.

To explore whether the production of crocetin could be improved via further increasing the copy number of *CCD2* and *ALDH* genes, plasmids pESC-LEU-*CCD2*-*CsALDH*, pESC-LEU-*CCD2*-*syaldh*, pESC-LEU2d-*CCD2*-*CsALDH*, and pESC-Leu2d-*CCD2-syaldh* were constructed (LEU: 20–30 copies per cell; LEU2d: 90–100 copies per cell; Erhart and Hollenberg, 1983) and transformed into TL005 and TL010 strains, respectively. Compared with the reference strains TL005 (33.09 ± 3.34 μg/g DCW) and TL0010 (29.47 μg/g DCW), the introduction of *CCD2-ALDH* on multi-copy plasmids only marginally increased crocetin production, with the highest (1.27-fold) achieved via the cloning of *CCD2-CsALDH* on the pESC-LEU2d plasmid. These results indicated that the benefits of multi-copy overexpression might be limited by the stability of the episomal plasmid system.

**Figure 3:**
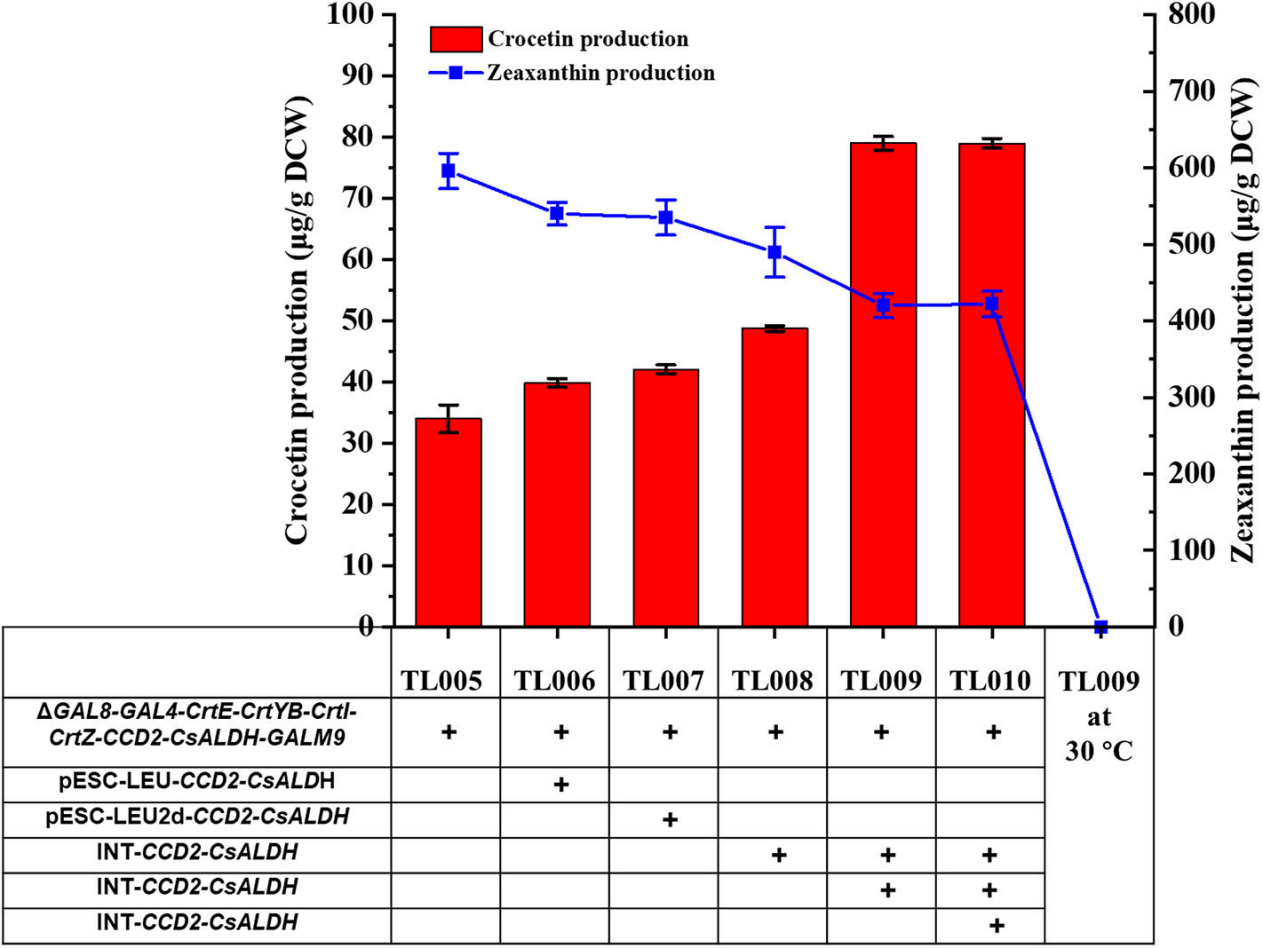
Enhancement of crocetin biosynthesis via optimizing the copy numbers of *CCD2* and Cs*ALDH* genes. With TL005 at 30°C as the reference strain, additional copies of *CCD2*-Cs*ALDH* were introduced either by multi-copy plasmids (TL006 and TL007) or multi-copy genome integration (TL008, TL009, and TL010). Crocetin (red bars) and zeaxanthin (blue squares) produced by the engineered strains were quantified by HPLC-MS. The strains were cultured at 30°C for 24 h and then switched to 24°C fermentation for additional 168 h. Error bars represent SD of biological triplicates.

Alternatively, *CCD2/CsALDH* and *CCD2/syaldh* expression cassettes were iteratively integrated into the chromosome of TL005 and TL011, respectively. Two chromosomal copies of *CCD2* and *ALDH* resulted in the construction of TL008 and TL0014, three copies for TL009 and TL015, and four copies for TL0010 and TL016, respectively (Table S2). Different with the plasmid bearing system, the production of crocetin in TL009 was significantly increased to 79.03 ± 1.78 μg/g DCW, which was approximately 2.38-fold higher than that in the reference strain TL005 (Figure 3). Interestingly, the introduction of *syaldh* failed to contribute to increasing crocetin production significantly (Figure S5). The discrepancy with the previous report (Chai et al., 2017) was probably due to the difference in the genetic background of the yeast host and the codon optimization algorithm. To further investigate the role of *syaldh* overexpression, strain TL017 without any heterologous *ALDH* was constructed and evaluated for crocetin production. Beyond expectation, strain TL017 and the *syaldh* overexpression strains produced comparable amount of crocetin, indicating that the endogenous ALDHs were active enough and the overexpression of *syaldh* didn't further increase the ALDH activities. Nevertheless, these results indicated the benefits of higher copy numbers and genetic stability in improving crocetin production.

### Optimization of Fermentation Conditions for Crocetin Production

**Figure 4:**
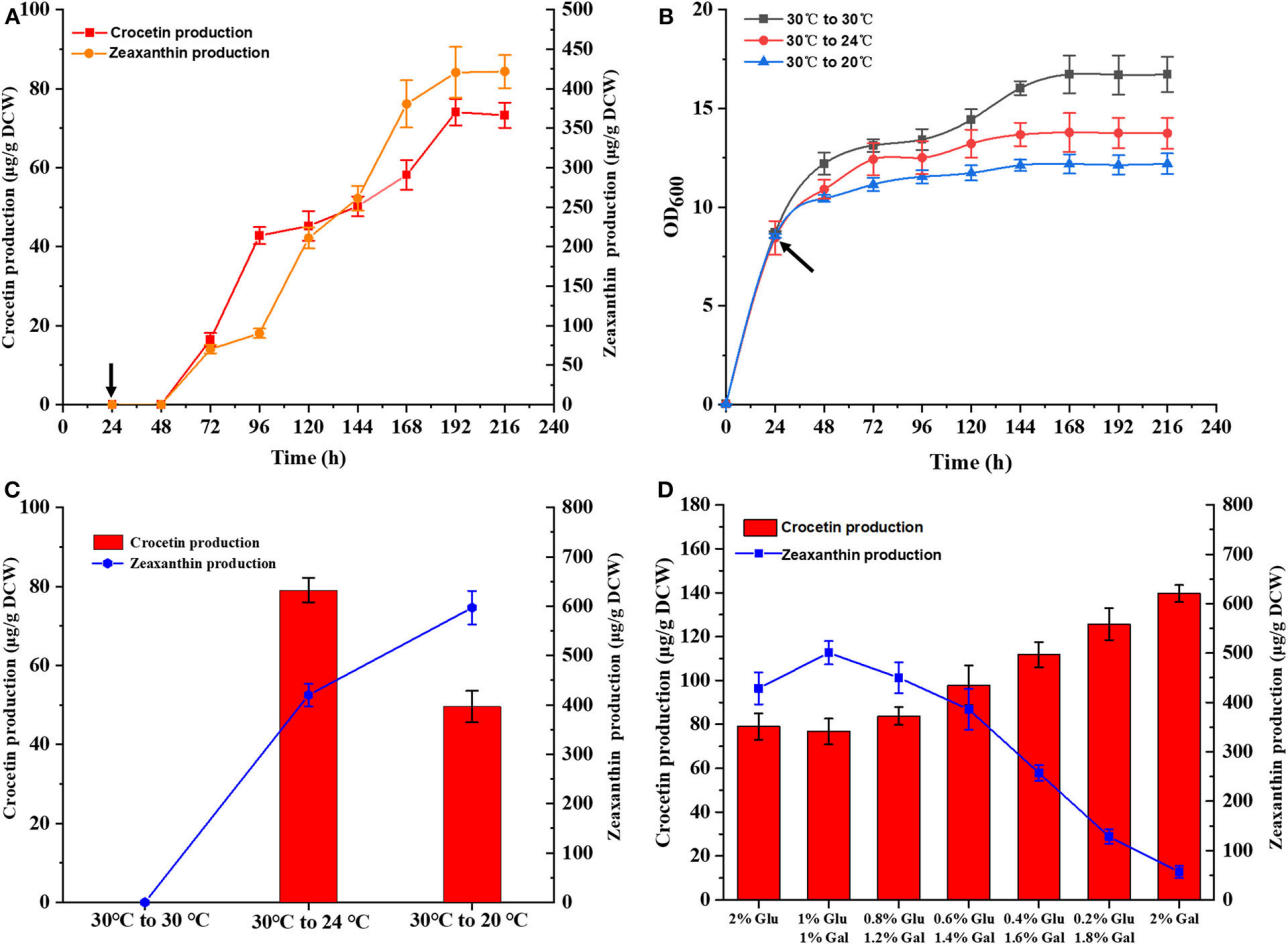
Optimization of fermentation conditions for crocetin production in the engineered strain TL009. **(A)** Time course of crocetin production with TL009 at 24°C. **(B)** Growth curves of the engineered yeast strain TL009 at 30°C (black squares), shifting to 24°C (red circles), and shifting to 20°C (blue triangles). Arrows indicated the timing of temperature shift. Error bars represent SD of biological triplicates. **(C)** Effect of fermentation temperatures (30°C, 30–24°C, 30–20°C) for zeaxanthin (blue line) and crocetin (red bars) biosynthesis. **(D)** Crocetin and zeaxanthin production of TL009 in the media with various ratios of glucose and galactose. Error bars represent SD of biological triplicates.

Subsequently, time-course studies on crocetin production was performed using strain TL009, containing three chromosomal copies of *CCD2* and *CsALDH*. TL009 was cultured in shake flasks at 30°C for 24 h and then continuously cultured for additional 192 h after shifting the temperature to 24°C. The target product crocetin started to accumulate at 72 h and reached the maximal production level (74.34 ± 2.31 μg/g DCW) in 192 h (Figures 4A,B). In accordance with above results, TL009 only accumulated crocetin at 24°C. The absence of any crocetin accumulation at 30°C (Figures 2B, 3) demonstrated the high sensitivity and stringency of the GAL4M9-mediated temperature-responsive regulation.

As mentioned above, the performance of the GAL regulon and enzymatic activity of CCD2 were largely dependent on temperature. Thus, the temperature for crocetin fermentation should be optimized. Strain TL009 was cultured in shake flasks at 30°C for 24 h and sub-cultured at 24 or 20°C for additional 192 h. Different with the previous result that 20°C was the optimal temperature for crocetin biosynthesis, strain TL009 produced a much higher level of crocetin at 24°C (79.03 ± 1.78 μg/g DCW) than that at 20°C (49.55 ± 1.24 μg/g DCW) (Figure 4C). Therefore, 24°C was more beneficial for converting zeaxanthin to crocetin in the temperature-responsive crocetin-producing yeast cell factory and used for the subsequent studies for crocetin production.

Finally, as the GAL regulon is tightly regulated by the composition of carbon sources, the ratio glucose and galactose supplemented to the fermentation media was optimized to further increase crocetin production by strain TL009. Generally, higher level production of crocetin was achieved with the increase of galactose composition (Figure 4D). More importantly, more zeaxanthin was converted and the zeaxanthin conversion yield was dramatically improved (Figure S6), indicating a balanced pathway for zeaxanthin biosynthesis and cleavage with galactose as the carbon source. Under the optimal conditions, strain TL009 produced crocetin at a level up to 139.67 ± 2.24 μg/g DCW in the present study, with a zeaxanthin conversion yield higher than 75%.

## Discussion

Crocetin have attracted many researcher's interests due to its biological activities, i.e., anti-tumor, enhancement of the rate of oxygen transport, and inhibition of pro-inflammatory mediators (Nam et al., 2010). With the goal of balancing zeaxanthin accumulation with temperature-regulated CCD2 activity, a temperature-responsive yeast cell factory for crocetin biosynthesis was established. The pigment formation and product analysis of the engineered strains after temperature shift indicated the high stringency and sensitivity of the temperature-regulated system. The introduction of additional copies of *CCD2-ALDH* further improved crocetin biosynthesis, with the chromosome-integrated strain (TL009) worked much better than the plasmid-bearing (TL006 and TL007) strains. These results demonstrated the advantages of using CRISPR-Cas9 technology to construct genetically stable and multi-copy integrated strains for efficient production of the desired products (Tyo et al., 2009; Shi et al., 2014).

Through the optimization of *CCD2-ALDH* copy numbers as well as shake-flask fermentation conditions, an efficient and stable crocetin-producing strain was established, with a titer of up to 139.67 ± 2.24 μg/g DCW and the highest zeaxanthin conversion yield of 77%. The crocetin production level was comparable to those reported in previous studies, ~160 μg/g DCW in 5-L bioreactors (Chai et al., 2017) and 62.79 μg/g DCW in shake flasks (Tan et al., 2019). Nevertheless, much higher zeaxanthin conversion yield (up to 77%) was achieved in the present study (Figure S6), highlighting the significance of synchronizing zeaxanthin biosynthesis and conversion for efficient crocetin biosynthesis as well as the advantage of the temperature-responsive system to achieve such a challenging goal. In addition, as the upper stream mevalonate pathway was systematically engineered to enhance terpenoid biosynthesis in the previous studies (Chai et al., 2017; Tan et al., 2019), the reported zeaxanthin production level was at least 10-fold higher than that achieved in the present study. As the direct precursor for crocetin biosynthesis, zeaxanthin biosynthesis should be strengthened, such as overexpression of the mevalonate pathway genes and the repression of the competing pathway genes, to further enhance crocetin production in the temperature-responsive yeast strain.

Although the production of crocetin was significantly improved via pathway engineering and/or metabolic engineering in both the present study and previous studies (Chai et al., 2017; Tan et al., 2019), zeaxanthin was still accumulated to relatively high levels, indicating zeaxanthin cleavage catalyzed by CCD2 as a rate-limiting step and room for further improving crocetin production. In other words, although the timing of zeaxanthin biosynthesis and cleavage was synchronized using the temperature switch, the pathway efficiency of zeaxanthin biosynthesis and crocetin biosynthesis should be further coordinated. Frusciante et al. found that CCD2 showed low affinity, low activity, and poor substrate specificity for zeaxanthin, which remained the biggest challenge for efficient production of crocetin using microbial cell factories. Increasing the catalytic activity of CCD2 by protein engineering or screening novel CCD2 enzymes, the fusion of CrtZ with CCD2 to channel the flux from zeaxanthin toward crocetin, and the engineering of the upper mevalonate pathway to enhance carotenogenesis are effective strategies to further improve crocetin production.

In summary, the present study reported the construction of a temperature-regulated crocetin production in *S. cerevisiae* for the first time, where the biosynthesis and cleavage of zeaxanthin was coordinated using an engineered GAL4-based temperature switch. Using the CRISPR-Cas9 based facile genome engineering technology, crocetin biosynthesis was optimized by integrating three copies of *CCD2* and Cs*ALDH* genes, with a final titer of 139.67 μg/g DCW and a zeaxanthin conversion yield of up to 77%. Our study provides a versatile platform for facilitating the biosynthesis of crocetin and other valuable epoxycarotenoids in yeast, such as violaxanthin, neoxanthin, and fucoxanthin (Cataldo et al., 2020).

## Data Availability Statement

All datasets generated for this study are included in the article/Supplementary Material.

## Author Contributions

TL, CD, MQ, and BZ performed the experiments. JL and TL conceived the study and wrote the manuscript. All authors read and approved the final manuscript.

## Conflict of Interest

The authors declare that the research was conducted in the absence of any commercial or financial relationships that could be construed as a potential conflict of interest.
