# Supplementary material for: Construction of a Stable and Temperature-Responsive Yeast Cell Factory for Crocetin Biosynthesis Using CRISPR-Cas9
Source: Front Bioeng Biotechnol. 2020 Jun 30;8:653. doi: 10.3389/fbioe.2020.00653 (PMC7339864; doi:10.3389/fbioe.2020.00653)
Supplement: Supplementary file 1 [file Presentation_1.pdf]

Supplementary Material

Supplementary Figures

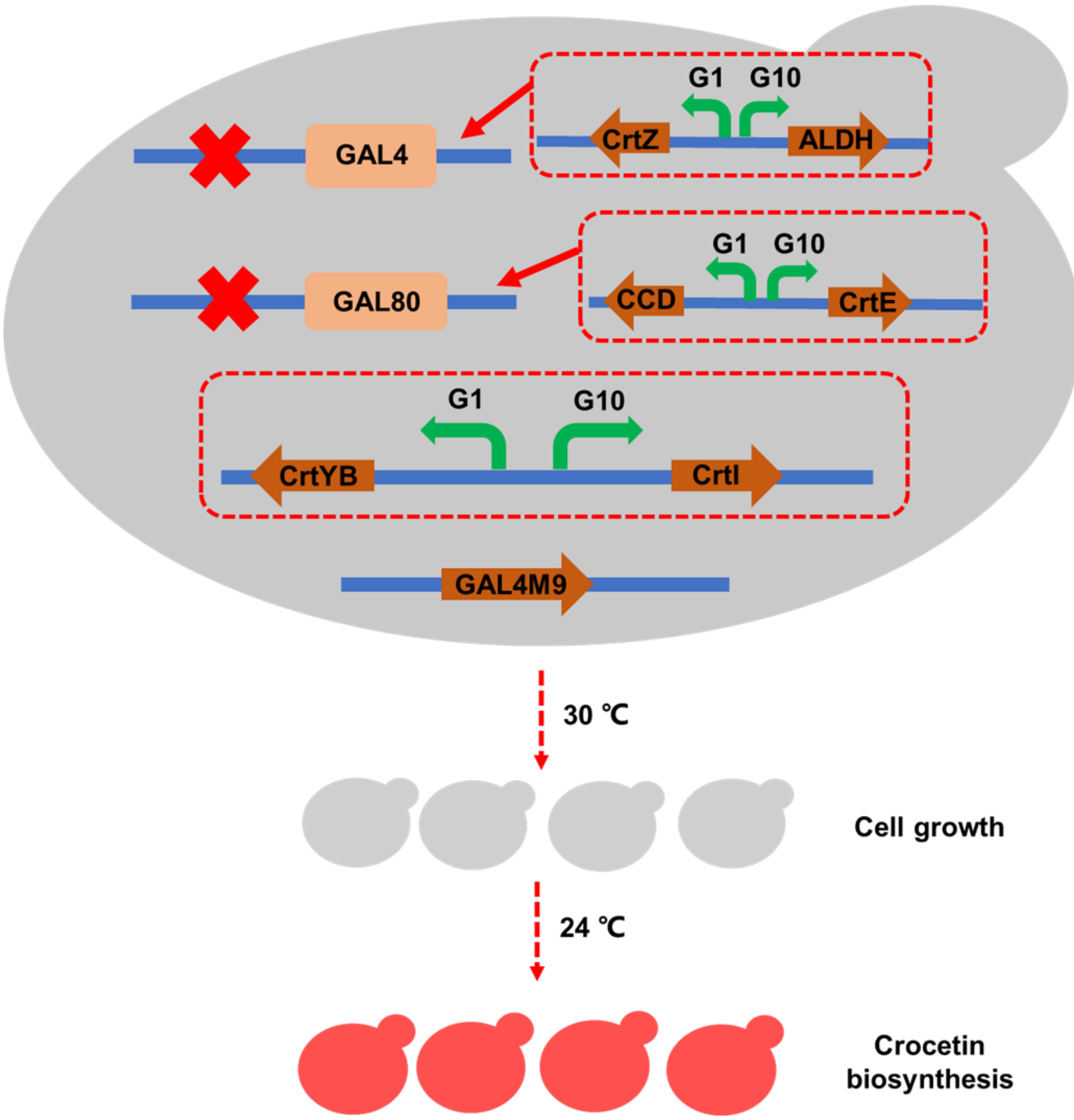

Supplementary Figure S1 Genome engineering of the GAL regulon for temperature-dependent crocetin biosynthesis, where zeaxanthin biosynthesis and cleavage were coordinately regulated using a GAL4M9-based temperature switch. *CrtZ* and *ALDH* expression cassettes were integrated into and replace the *GAL4* locus; *CCD* and *CrtE* expression cassettes were integrated into and replace the *GAL80* locus; *CrtYB* and *CrtI* expression cassettes were integrated into the chromosome locus at ChrXIV: 298,004-298,023; *GAL4M9* expression cassette was integrated into the chromosome locus at ChrIV: 56,137-56,156. G1, *GAL1* promoter; G10, *GAL10* promoter ; GAL4, transcription factor for activating GAL regulon genes; GAL4M9, GAL4 mutant showing low temperature induction; GAL80, transcriptional regulator involved in the repression of GAL genes by inhibiting GAL4; CrtE, lycopene ε-cyclase; CrtYB, bifunctional phytoene synthase and lycopene cyclase; CrtI, phytoene desaturase; CrtZ, β-carotene hydroxylase; CCD, carotenoid cleavage dioxygenase; ALDH, aldehyde dehydrogenase

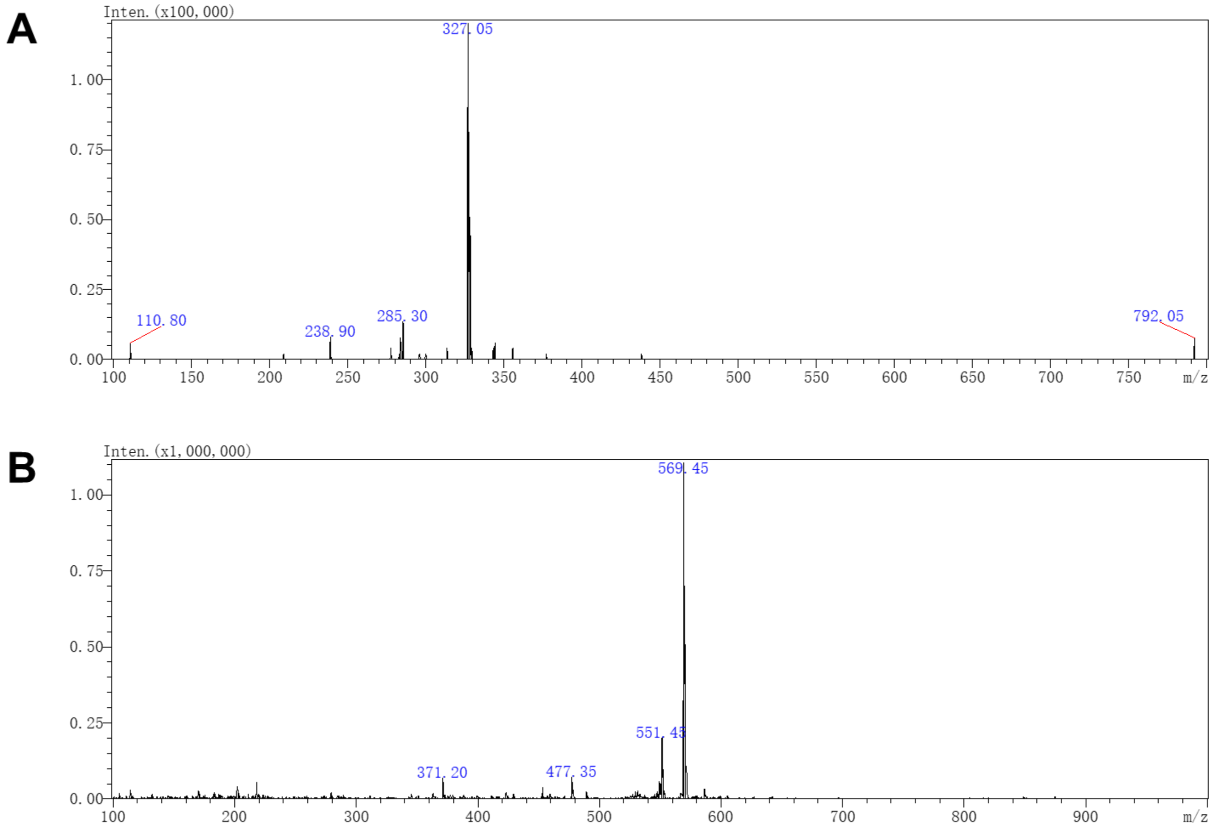

Supplementary Figure S2 HPLC-MS chromatograms of the crocetin and zeaxanthin standards. (A) MS spectra of crocetin standard. (B) MS spectra of zeaxanthin standard

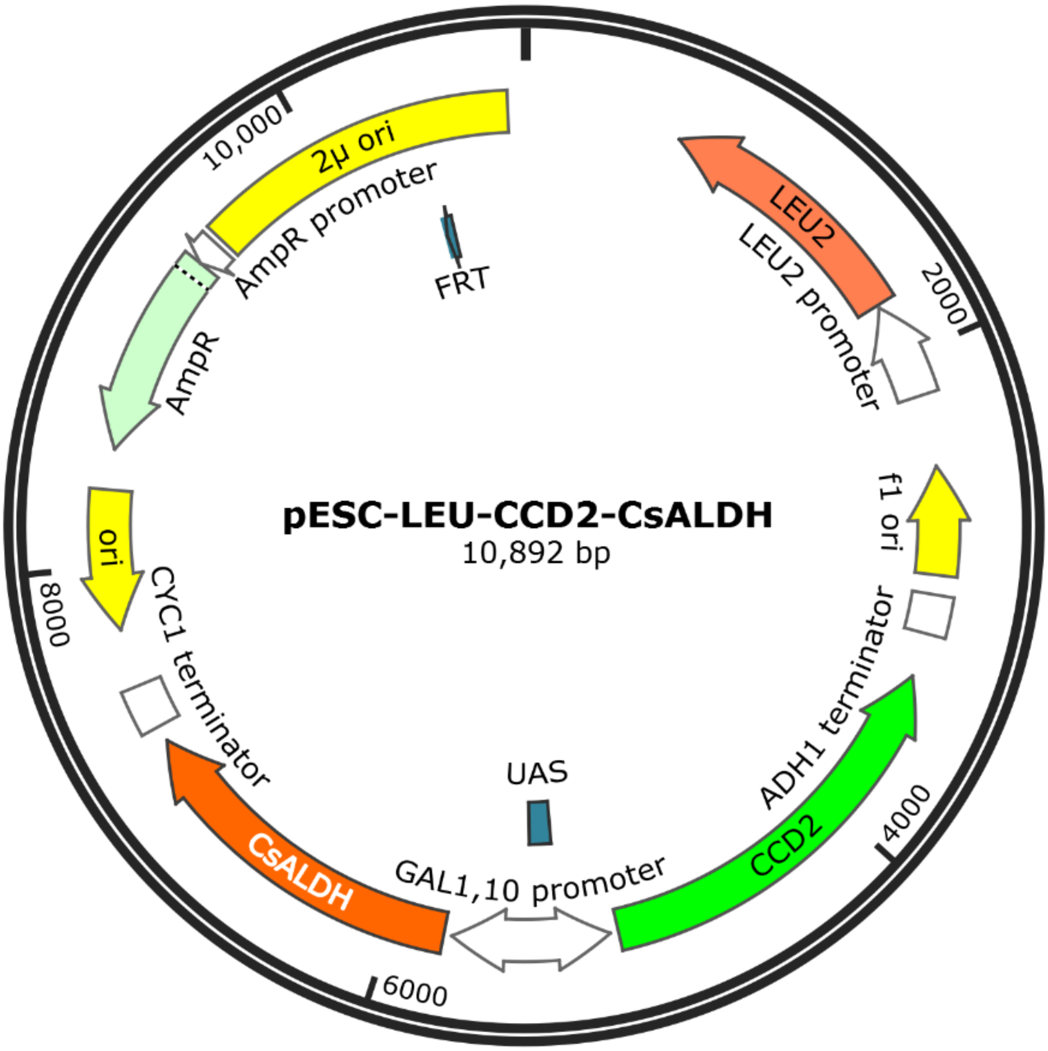

Supplementary Figure S3 The plasmid map of pESC-LEU-*CCD2-CsALDH*. *CCD2* was cloned into the *Bam*HI/*Xho*I sites of MCS1 and *CsALDH* was cloned into the *Not*I site of MCS2

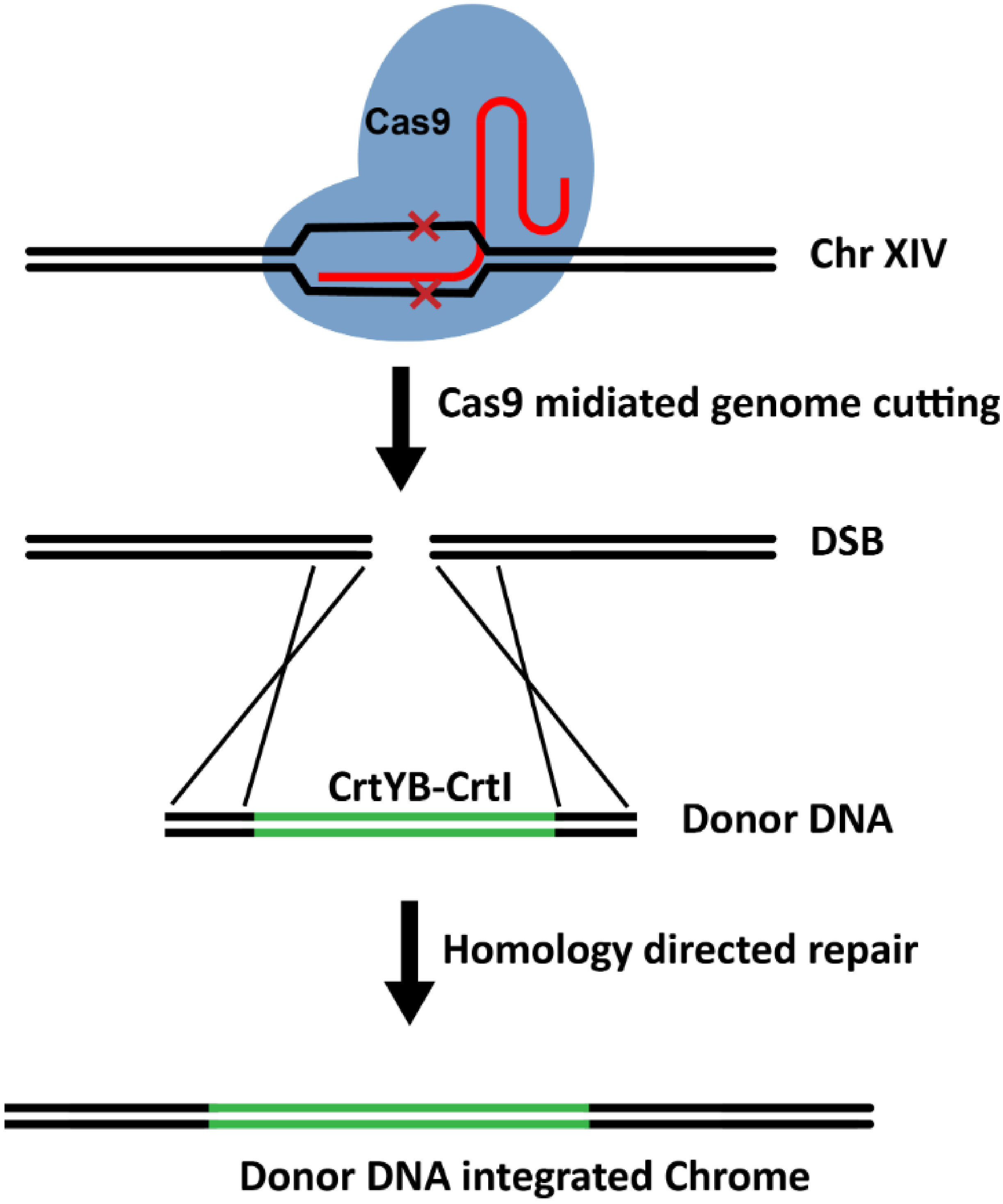

Supplementary Figure S4 A general scheme for the integration of heterologous gene expression cassettes (with *CrtYB* and *CrtI* expression cassettes shown as an example) into the genome of *S. cerevisiae*. The corresponding gRNA sequences and chromosomal integration loci were provided in Table S4

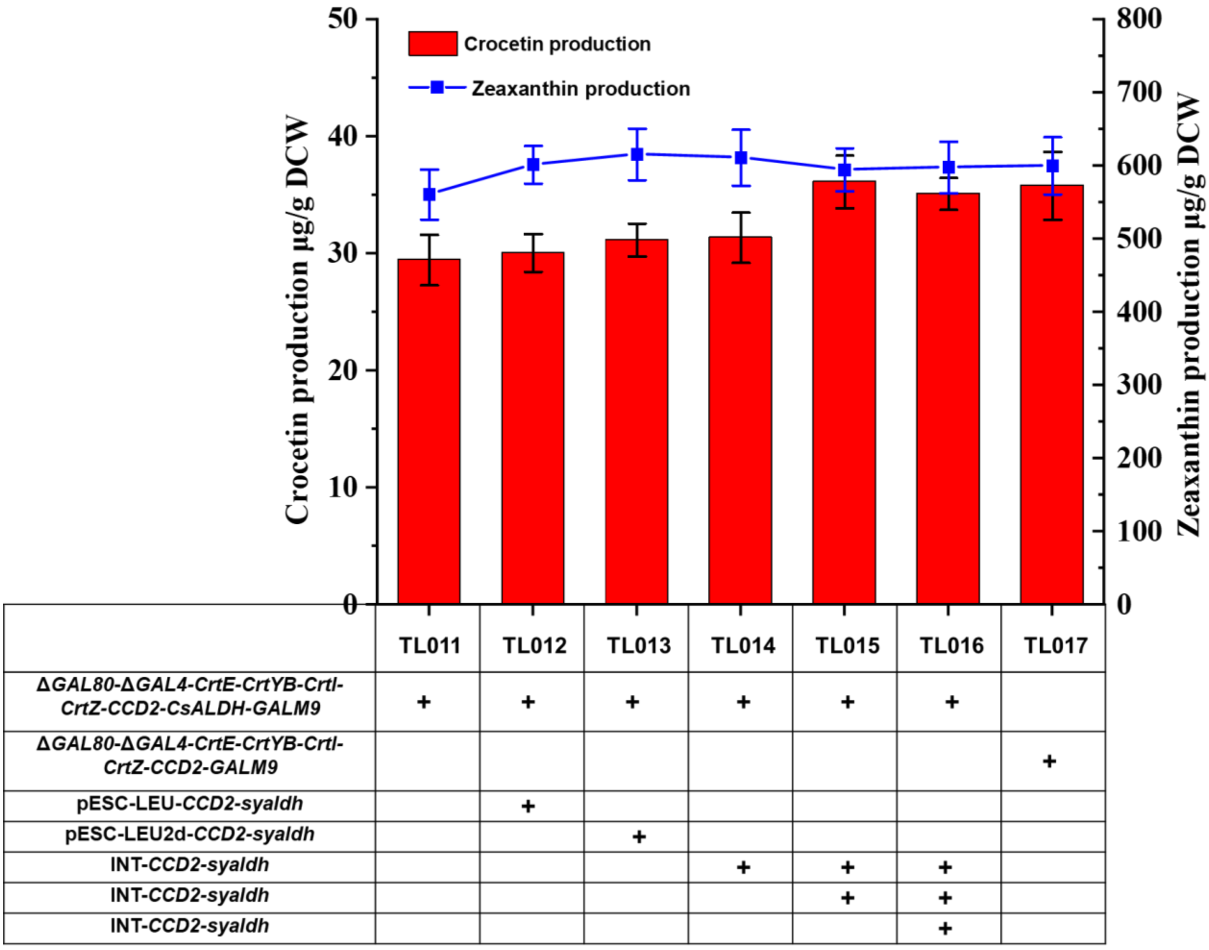

	TL011	TL012	TL013	TL014	TL015	TL016	TL017
ΔGAL80-ΔGAL4-CrtE-CrtYB-CrtI-CrtZ-CCD2-CsALDH-GALM9	+	+	+	+	+	+	
ΔGAL80-ΔGAL4-CrtE-CrtYB-CrtI-CrtZ-CCD2-GALM9							+
pESC-LEU-*CCD2-syaldh*		+					
pESC-LEU2d-*CCD2-syaldh*			+				
INT-*CCD2-syaldh*				+	+	+	
INT-*CCD2-syaldh*					+	+	
INT-*CCD2-syaldh*						+	

Supplementary Figure S5 Effect of additional copies of *CCD2* and *syaldh* on the production of crocetin. With TL011 as the reference strain, additional copies of *CCD2-syaldh* were introduced either by multi-copy plasmids (TL012 and TL013) or multi-copy genome integration (TL015 and TL016). A *syaldh*-free strain (TL017) was constructed to evaluate the role of *syaldh* overexpression on crocetin production. The strains were cultured at 30 °C for 24 h and then switched to 24 °C fermentation for additional 168 h. Error bars represent SD of biological triplicates.

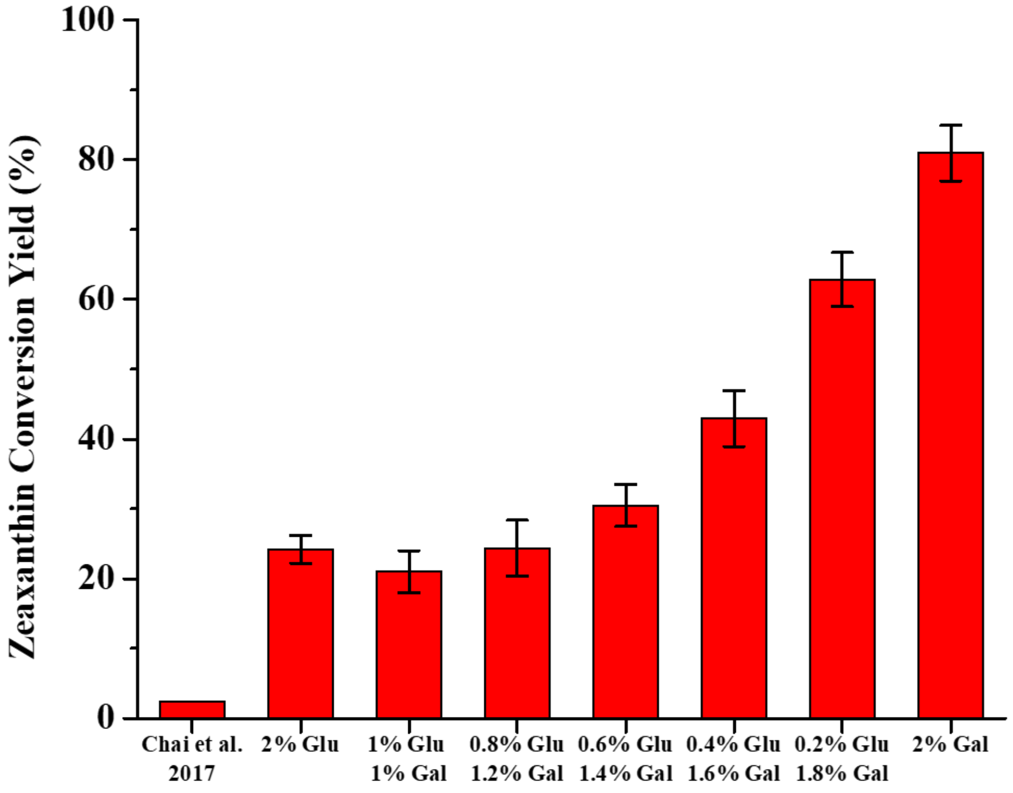

Supplementary Figure S6 Comparison of zeaxanthin conversion yield of the temperature-responsive cell factories with a previous study for crocetin production. The conversion yield of the previous study (Chai et al., 2017) was calculated for strain SyBE Sc0123C009 cultured in shake flasks at 20 °C, where both crocetin and zeaxanthin production was provided. Error bars represent the standard deviations of three replicates

Supplementary Tables

Supplementary Table S1 DNA sequences of genes used in this study

Genes	DNA sequences
CCD2	ATGGCAAACAAAGAAGAAGCGGAAAAACGTAAAAAGAAACCGAAACCGCTGAAAGTGCTGATTAC<br>GAAAGTGGACCCGAAACCGCGTAAAGGTATGGCGAGCGTTGCGGTGGACCTGCTGGAAAAAGCAT<br>TTGTGTATCTGCTGAGCGGAAATAGCGCCGCCGATCGTAGCAGTAGCAGCGGCCGCCGTCGTCGTAA<br>AGAACATTATTATCTGAGTGGTAATTACGCTCCTGTTGGTCATGAAACCCCGCCGAGCGATCATCTGC<br>CGATCCACGGTAGCCTGCCTGAATGTCTGAATGGTGTTTTTCTGCGTGTGGGGCCGAATCCGAAATT<br>TGCGCCGGTTGCAGGTTATAATTGGGTGGACGGTGATGGAATGATTCATGGTCTGCGTATTAAAGATG<br>GTAAAGCCACCTACCTGAGTCGTTATATTAAAACAAGCCGTTTTAAACAGGAGGAATATTTTGGTCG<br>CGCGAAATTTATGAAAATTGGTGATCTGCGTGGTCTGCTGGGCTTCTTTACAATCCTGATCCTGGTTC<br>TGCGTACCACCCTGAAAGTTATTGATATCAGCTATGGACGTGGTACAGGTAATACGGCGCTGGTGTAT<br>CATAATGGTCTGCTGCTGGCTCTGTCTGAGGAAGACAAACCGTATGTTGTTAAAGTTCTGGAAGATG<br>GTGATCTGCAGACCCTGGGAATTTTAGACTATGACAAAAAGCTGAGTCATCCTTTTACAGCCCATCC<br>TAAAATCGATCCGCTGACCGATGAAATGTTTACCTTCGGTTATAGCATTAGCCCGCCTTATCTGACCTA<br>TCGTGTTATTTCCAAAGACGGAGTTATGCAAGATCCGGTCCAGATTTCCATTACCAGCCCTACCATTA<br>TGCATGACTTTGCAATTACAGAAAACTATGCAATTTTCATGGACCTGCCGCTGTATTTTCAGCCGGAA<br>GAAATGGTTAAAGGTAAATTTGTTAGCAGCTTCCATCCGACCAAACGCGCCCGCATTGGTGTTCTGC<br>CACGTTATGCCAAAGACGAGCACCCGATTCGTTGGTTTGATCTGCCTAGTTGTTTTATGACCCATAAT<br>GCCAATGCATGGGAGGAAAATGATGAAGTTGTTCTGTTTACGTGTCGTCTGGAAAGCCCGGATCTGG<br>ATATGCTGAGCGGTCCGGCAGAAGAAGAAATTGGGAATAGCAAAAGCGAACTGTATGAAATGCGTT<br>TTAATCTGAAAACCGGGATCACCAGCCAGAAACAGCTGTCAGTTCCGTCAGTGGACTTTCCACGTAT<br>TAATCAGAGCTATACGGGTCGCAAACAACAGTATGTTTATTGTACCCTGGGTAATACCAAAATTAAAG<br>GTATTGTTAAATTCGACCTGCAGATTGAACCGGAAGCAGGTAAAACCATGCTGGAAGTTGGTGGTAA<br>TGTTCAGGGCATTTTTGAGCTGGGGCCGCGTCGTTATGGCAGCGAAGCAATTTTTGTGCCGTGTCAG<br>CCTGGTATTAAATCTGATGAAGATGATGGCTACCTGATTTTTTTTGTTCATGATGAAAACAACGGCAA<br>ATCTGAAGTTAATGTTATCGATGCAAAAACCATGAGCGCGGAACCGGTTGCAGTTGTGGAGCTGCCG<br>AGCCGTGTGCCGTATGGTTTTCATGCACTGTTTCTGAATGAAGAAGAACTGCAGAAACATCAGGCCG<br>AAACCTAA
CsALDH	ATGGCATTTGATGGTGAAAAAGCGAAAGAAATGGTGAAAGAACTGCGTGAAAGCTTTAATAAAGG<br>TACGACGCGCAGCTATGAATGGCGCATGAAACAGCTGAAAGCGATGGAAAAAATGACCGAAGAA<br>AAAGAAAAAGATATTATGGATGCACTGGAAAGCGATCTGAGCAAACCGCAGCTGGAAAGCTTTCT<br>GCATGAAATTAGCATGGCAAAAAGCGTTTGTCAGTTTGCAGCAAAAAATCTGAAACGTTGGATGA<br>AACCGGAAAAAGTTCCGGCACAGCTGACCACCTTTCCGAGCGTTGGTAATATTGTTGCAGAACCG<br>TTTGGTGTTGTTCTGATTATTAGCGCATGGAATTTTCCGTTTCTGCTGAGCCTGGAACCGGTTATTG<br>GTGCAATTGCAGCAGGTAATACCGTTGTTCTGAAACCGAGCGAAATTGCACCGGCAACCAGCAGC<br>CTGTTTGCACGTATTCTGCTGGAATATGTTGATACCAGCTGTGTTCGTGTTGTTGAAGGTGCAGTTC<br>CGGAAACCACCGCACTGCTGGAACAGAAATGGGATAAAATTTTTTATACCGGTAATGGTAAAGTTG<br>GTCGTGTTGTTATGGCAGCAGCAGCAAAACATCTGACCCCGGTTGTTCTGGAACTGGGTGGTAAAT<br>GTCCGGTTGTTGTTGATAGCAATATTGATCTGAAAGTTGCAACCAAACGTGTTGTTGTTGGTAAATG<br>GGGTTGTAATAATGGTCAGGCATGTATTGCACCGGATTATATTATTACCACCAAAAGCTTTGCACCG<br>AAACTGGTTGAAAGCCTGAAAATTACCCTGGAACGTTTTTATGGTGAAGATCCGCTGGAAACCGA

	AGATCTGAGCCGTATTGTTAATGAAAATCATGTTGCACGTCTGGCACGTCTGCTGGATGATGATATG
	GTTAGCGGTAAAATTATTTATGGTGGTAAACGTGATGAAAAACGTCTGAAAATTGCACCGACCCTG
	CTGCTGGATGTTCCGGATGATAGCCTGATTATGAAAGAAGAAATTTTTGGTCCGCTGCTGCCGATTA
	TTACCGTTGATAAAATTGAAGATAGCTTTGCAGTTATTAATAGCAAAACCAAACCGCTGGCAGCATA
	TCTGTTTACCAAAAATAAAAATCTGGAACGTATGTTTGTTGAAACCGTTAGCAGCGGTGGTATGCT
	GATTAATGATACCGTTCTGCATGTTGCAAATCCGTATCTGCCGTTTGGTGGTGTTGGTGAAAGCGGT
	ACCGGTAGCTATCATGGTAAATTTAGCTTTAATGCATTTAGCCATAAAAAAGCAGTTCTGAGCCGTG
	GTTTTGGTGGTGAAGTTGGTGCACGTTATCCGCCGTATACCGATAAAAAACGTAAAATTATTCGTGC
	ACTGCTGGCAGGTAATATTATTGCACTGGTTCTGGCATTTTTTGGTTTTAGCAAAAGCTAA
syaldh	ATGAACACCGCCAAAACCGTTGTGGCCGAACAGCGTGACTTTTTTCGCCAGGGGAAAACCAAAA
	GCGTGCAGGACCGCCTGACCGCACTGGCCAAACTGAAAACCCAGATCCAGGCCCAGGAAGAAGA
	AATCATTAAAGCACTGAAACAAGATTTCGGCAAACCAACCTTTGAAAGCTATGTTAATGAAATTCT
	GGGTGTTATTCGTGAAATCAATTACTATCAAAAACACCTGCAACAGTGGAGCAAACCGCAGCGTGT
	TGGAACCAATCTGATGGTGTTTCCGGCAAGCGCACAGCTGCGTCCAGAGCCTCTGGGTGTTGTTCT
	GATTATCTCTCCTTGGAATTACCCTTTTTATCTGTGTCTGATGCCGCTGATTGGAGCAATCGCCGCCG
	GTAATTGTGTTGTTGTTAAACCGTCAGAATATACCCCTGCAATTAGTGGTGTTATCACCCGTCTGATT
	CAGAATGTTTTTTCTCCTGCCTGGGCAACCGTTGTTGAAGGTGATGAGACAATCAGTCAGCAGCTG
	CTGCAGGAAAAATTTGACCATATTTTTTTCACCGGTAGCCCGCGTGTTGGCCGTCTGATTATGGCAG
	CAGCAGCCGAACAGCTGACCCCGGTTACCCTGGAACTGGGCGGTAAAAGCCCTTGTGTTGTTGAT
	CGTGAAATTAATCTGCAGGAAACCGCAAAACGTATTATGTGGGGCAAACTGGTAAATGCAGGACA
	GACCTGTGTTGCACCGGATTATCTGCTGGTTGAACAGTCTTGTCTGGAGCAGCTGCTGCCTGCCCT
	GCAGCAGGCGATTCAAATGCTGTTCGGTGAAAATCCTGCGCACAGTCCGGATTATACGCGTATTGT
	TAATCAGCAACAATGGAGCCGTCTGGTTAGCCTGCTGAGCCATGGTAAAGTTATTACCCGTGGCGA
	TCATAATGAAGGTGATCGTTATATTGCACCGACCCTGATTATTGATCCGGATCTGAATAGCCCGCTGA
	TGCAAGAAGAGATTTTCGGTCCGATTCTGCCGATTCTGACCTATCAAAGCCTGAGCGAAGCAATTG
	ATTTTATTAATATCAAACCTAAACCTCTGGCACTGTATTTTTTCTCTAATAATCGTCAGAAACAGGAG
	GAAATTCTGCAAAGCACCTCCAGCGGTAGCGTGTGTCTGAATGATATTCTGCTGCACCTGACCGTG
	ACCGACCTGCCTTTTGGTGGTGTGGGCGAAAGCGGTATGGGTCGCTATCATGGGAAAGCAACATTT
	GACACCCTGAGTAATTATAAAAGCATCCTGCGTCGTCCTTTTTGGGGCGAAACCAATCTGCGTTATA
	GTCCGTATGGTAAAAAGATGAACCTGATTAAAAAGCTGTTCAGTCTCGAGTAA

Supplementary Table S2 Strains and plasmids used in this study

Plasmid/strain name	Description	Source
**Plasmids**		
pRS41K-SpCas9	CEN/ARS; G418; AmpR; *TEF1p-SpCas9-TEF1t*	Lian J et al., 2017
p423-SpSgH	2μ; *HIS3*; *AmpR*; *SNR52p-SUP4t*	Lian J et al., 2017
p426-SpSgH	2μ; *URA3*; *AmpR*; *SNR52p-SUP4t*	Lian J et al., 2017
pESC-URA	2μ; *URA3*; *AmpR*; *GAL1p*-MCS1-*CYC1t*; *GAL10p*-MCS2-*ADH1t* (NCBI Accession AF063585)	Agilent Technologies, Inc.
pESC-LEU	2μ; *LEU2*; *AmpR*; *GAL1p*-MCS1-*CYC1t*; *GAL10p*-MCS2-*ADH1t* (NCBI Accession AF063849)	Agilent Technologies, Inc
pESC-LEU2d	2μ; *LEU2*d; AmpR; *GAL1p*-MCS1-*CYC1t*; *GAL10p*-MCS2-*ADH1t* (Addgene plasmid #20120)	Ro DK et al., 2006
pUMRI-P$_{ACT1}$-*GAL4M9*	loxP-KanMX-URA3-pBR322ori-loxP, T$_{ADH1}$-*GAL4M9*-P$_{ACT1}$-MCS2-T$_{CYC1}$, LPP1 homologous arm	Zhou, P et al.,2018
pESC-URA-*CCD2-CrtE*	2μ; *URA3; AmpR; GAL1p-CCD2-CYC1t; GAL10p-CrtE-ADH1t*	This study
pESC-URA-*CrtYB-CrtI*	2μ; *URA3; AmpR; GAL1p-CrtYB-CYC1t; GAL10p-CrtI-ADH1t*	This study
pESC-URA-*CrtZ-CsALDH*	2μ; *URA3; AmpR; GAL1p-CrtZ-CYC1t; GAL10p-CsALDH-ADH1t*	This study
pESC-URA-*CrtZ-syaldh*	2μ; *URA3; AmpR; GAL1p-CrtZ-CYC1t; GAL10p-syaldh-ADH1t*	This study
pESC-LEU-*CCD2-CsALDH*	2μ; *LEU2; AmpR; GAL1p-CrtZ-CYC1t; GAL10p-CsALDH-ADH1t*	This study
pESC-LEU-*CCD2-syaldh*	2μ; *LEU2; AmpR; GAL1p-CrtZ-CYC1t; GAL10p-syaldh-ADH1t*	This study
pESC-LEU2d-*CCD2-CsALDH*	2μ; *LEU2*d; *AmpR; GAL1p-CrtZ-CYC1t; GAL10p-CsALDH-ADH1t*	This study
pESC-LEU2d-*CCD2-SyALDH*	2μ; *LEU2*d; *AmpR; GAL1p-CrtZ-CYC1t; GAL10p-SyALDH-ADH1t*	This study
**Strains**		
BY4741	MATa: *his3*Δ1 *leu2*Δ0 *met15*Δ0 *ura3*Δ0	ATCC
TL001	BY4741 with pRS41K-SpCas9	This study
TL002	TL001-Δ*GAL80::GAL1p-CCD2-CYC1t-GAL10p-CrtE-ADH1t*	This study
TL003	TL002-*GAL1p-CrtYB-CYC1t-GAL10p-CrtI-ADH1t*	This study
TL004	TL003-P$_{ACT1}$-*GAL4M9*- T$_{ADH1}$	This study
TL005	TL004-Δ*GAL4*::*GAL1p-CrtZ-CYC1t-GAL10p-CsALDH-ADH1t*	This study
TL006	TL005/pESC-LEU-*CCD2-CsALDH*	This study
TL007	TL005/pESC-LEU2d-*CCD2-CsALDH*	This study
TL008	TL005-*GAL1p-CCD2-CYC1t-GAL10p-CsALDH-ADH1t*	This study
TL009	TL008-*GAL1p-CCD2-CYC1t-GAL10p-CsALDH-ADH1t*	This study
TL010	TL009-*GAL1p-CCD2-CYC1t-GAL10p-CsALDH-ADH1t*	This study
TL011	TL004-Δ*GAL4*::*GAL1p-CrtZ-CYC1t-GAL10p-SyALDH-ADH1t*	This study
TL012	TL011/pESC-LEU-*CCD2-syaldh*	This study
TL013	TL011/pESC-LEU2d-*CCD2-syaldh*	This study
TL014	TL011-*GAL1p-CCD2-CYC1t-GAL10p-syaldh-ADH1t*	This study
TL015	TL014-*GAL1p-CCD2-CYC1t-GAL10p-syaldh-ADH1t*	This study
TL016	TL015-*GAL1p-CCD2-CYC1t-GAL10p-SyALDH-ADH1t*	This study
TL017	TL004-ΔGAL4::*GAL1p-CrtZ-CYC1t*	This study

Supplementary Table S3 List of primers used in this study

Primer name	Sequence (5′-3′)
**For plasmids construction**	
CrtYB-BamHI-F	AACCCCGGATCCATGACGGCTCTCGCATATTA
CrtYB-XhoI-R	GCTTACTCGAGTTACTGCCCTTCCCATCCGC
CrtI-NotI-F	CGTCATCCTTGTAATCCATCGATACTAGTTCAGAAAGCAAGAACACCAAC
CrtI-NotI-R	CGAATTCAACCCTCACTAAAGGGCGGCCGCATGGGAAAAGAACAAGATCA
CCD2-BamHI-F	AACCCCGGATCCATGGCAAACAAAGAAGAAGC
CCD2-XhoI-R	GCTTACTCGAGTTAGGTTTCGGCCTGATGTT
CrtE-NotI-F	TCTGGCGAAGAATTGTTAATTAAGAGCTCTCACAGAGGGATATCGGCTAG
CrtE-NotI-R	AACCCTCACTAAAGGGCGGCCGCACTAGTATGGATTACGCGAACATCCT
CrtZ-BamHI-F	AACCCCGGATCCATGTTGTGGATTTGGAATGC
CrtZ-XhoI-R	GCTTACTCGAGTTACTTCCCGGATGCGGGCTC
CsALDH-NotI-F	TTGTAATCCATCGATACTAGTGCGGCCGCTTAGCTTTTGCTAAAACCAA
CsALDH-NotI-R	AACCCTCACTAAAGGGCGGCCGCACTAGTATGGCATTTGATGGTGAAAAAG
SyALDH-NotI-F	TTGTAATCCATCGATACTAGTGCGGCCGCTTACTCGAGACTGAACAGCTTTT
SyALDH-NotI-R	AAGTAAGAATTTTTGAAAATTCGAATTCATGAACACCGCCAAAACCGTT
**For genome integration**	
ΔGAL80-INT-CCD2-CrtE-F	CAATCTCGATAGTTGGTTTCCCGTTCTTTCCACTCCCGTCTTCAGGCTGCGCAACTGTT
ΔGAL80-INT-CCD2-CrtE-R	CGTTCGCTGCACTGGGGGCCAAGCACAGGGCAAGATGCTTAGTGAGCTGATACCGCTCG
INT-CrtYB-CrtI-F	CTATGTGACGCTGTGTATTCTTTGTTGTAGTTATGCTCCATTCAGGCTGCGCAACTGTT
INT- CrtYB-CrtI-R	TGTACGCTATACATTTACGTGCTGAGCTCCTAGGAAAGCTAGTGAGCTGATACCGCTCG
ΔGAL4-INT-CrtZ-ALD-F	GAAGTGAACTTGCGGGGTTTTTCAGTATCTACGATTCATTTTCAGGCTGCGCAACTGTT
ΔGAL4-INT-CrtZ-ALD-R	ATCATTTTAAGAGAGGACAGAGAAGCAAGCCTCCTGAAAGAGTGAGCTGATACCGCTCG
INT-GAL4M9-F	GTTTTCTTATTTCTTTCTTTTTAAAAAACTTTCTTAATATTGTAGGCCTCTTCGCTATT
INT-GAL4M9-R	TTGAGAAAAAAAGTGTATATCATTACATTACTTTACACCAAGTCGTATTACGGATCCTC
INT- ALDH-F(TL008 ,014)	ATTGTAGTGCAGAAGGTAACAGCAAAAACAAATAGTTCACGAGCGACCTCATGCTATAC
INT- ALDH-R(TL008,014)	GAATTAGTTTATTAGAGGAAGTGCCAGGCGACATAAAGTTCTTCGAGCGTCCCAAAACC
INT- ALDH-F(TL009,015)	TCTAGTATCAAAGAAACTTACTATGACGCAGTTTAGGATCGAGCGACCTCATGCTATAC
INT- ALDH-R(TL009,015)	GACACTGAATAAACAAGGGGCTTTACGATGGAGTAGTAGACTTCGAGCGTCCCAAAACC
INT- ALDH-F(TL010,016)	TACTGTTGATTGTTCGTTTATTTGTATAATTGAGTTTACAGAGCGACCTCATGCTATAC
INT- ALDH-R(TL010,016)	TTATAAGTTTGCTTTTTGTCACTCTCTTGGCCCTAATTACCTTCGAGCGTCCCAAAACC

* Restriction sites are underlined.

Supplementary Table S4 The sequences of SgRNAs in this study

SgRNA Name	Sequence (5′-3′)	Chromosome locus
Sg-ΔGAL80	GATAGTGATAGCTATCCAAG	ChrXIII: 171,860-171,879
Sg-ΔGAL4	TTCTGTCGCGATATACACAG	ChrXVI: 81,346-81,365
Sg-INT- CrtYB-CrtI	CGATACAGTGGCTGCTCATG	ChrXIV: 298,004-298,023
Sg-INT-GAL4M9	ACACGTTTGTGGTTATAAGG	ChrIV: 56,137-56,156
Sg-INT- CCD2-ALDH (for TL 008 and TL014)	TAAAGCCACCACATCGCAAA	ChrXVII: 562,256-562,275
Sg-INT- CCD2-ALDH (for TL009 and TL015)	CCTGTGGTGACTACGTATCC	ChrXIV: 262,867-262,886
Sg-INT- CCD2-ALDH (for TL0010 and TL016)	CAACAATTGTTACAATAGTA	ChrVII: 508,439-508,458

Supplementary References

Chai, F., Wang, Y., Mei, X., Yao, M., Chen, Y., Liu, H., et al. (2017). Heterologous biosynthesis and manipulation of crocetin in *S. cerevisiae*. *Microbial Cell Factories*. 16:1, 54. doi: 10.1186/s12934-017-0665-1

Lian, J., Hamedirad, M., Hu, S., Zhao, H. (2017). Combinatorial metabolic engineering using an orthogonal tri-functional CRISPR system. *Nat Commun*. 8:1,1688. doi: 10.1038/s41467-017-01695-x

Ro, D. K., Paradise, E. M., Ouellet, M., Fisher, K. J., Newman, K. L., Ndungu, J. M., et al. (2006). Production of the antimalarial drug precursor artemisinic acid in engineered yeast. *Nature* 440, 940-943. doi: 10.1038/nature04640

Zhou, P., Xie, W., Yao, Z., Zhu, Y., Ye, L., Yu, H. (2018). Development of a temperature-responsive yeast cell factory using engineered GAL4 as a protein switch. *Biotechnol. Bioeng*. 115:5,1321-1330. doi: 10.1002/bit.26544
